# A Case of Postmenopausal Uterine Adenosarcoma With Concomitant Diabetes Mellitus

**DOI:** 10.7759/cureus.78978

**Published:** 2025-02-14

**Authors:** Haruko Fujito, Tomoyo Yasui, Yuichiro Awazu, Kazuharu Tanaka, Kenta Oue, Takuma Wada, Reiko Tasaka, Makoto Yamauchi, Takeshi Fukuda, Toshiyuki Sumi

**Affiliations:** 1 Obstetrics and Gynecology, Izumiotsu Women's and Children's Hospital, Izumiotsu, JPN; 2 Obstetrics and Gynecology, Osaka Metropolitan University Graduate School of Medicine, Osaka, JPN

**Keywords:** abnormal vaginal bleeding, diabetes mellitus, polypoidal mass, postmenopausal woman, uterine adenosarcoma

## Abstract

Uterine adenosarcoma is a rare, low-grade malignant tumor that usually arises from the endometrium and presents as a large polypoid mass occupying the endometrial cavity. A correct histopathological diagnosis of adenosarcoma from a small specimen is often difficult, and pre-operative MR imaging findings are generally not specific enough to establish an adenosarcoma diagnosis.

A 60-year-old nulliparous woman was referred to our hospital who presented with vaginal bleeding. Her medical history was notable for type 2 diabetes mellitus and hypertension. Upon pelvic examination, a large endometrial polyp prolapsing through the cervix and vagina was observed while she was bleeding profusely. We performed a biopsy on the polyp, but the pathological diagnosis showed no malignancy. On MR imaging, the tumor presented both low and high signal intensities on T2WI, low signal intensity on T1WI, and a slightly high DWI signal intensity. Therefore, a prolapsed leiomyoma was suspected, and a hysteroscopic tumor excision was performed. The pathological examination found that the tumor was a uterine adenosarcoma. We thereafter performed an abdominal total hysterectomy with a bilateral salpingo-oophorectomy. The final diagnosis was a uterine adenosarcoma, T1aN0M0. In postmenopausal women with diabetes, where the pathological biopsy findings and imaging of a polypoidal mass with abnormal vaginal bleeding do not lead to a suspected malignant neoplasm, it is still important to remove the tumor and examine it in order to attain a current diagnosis because it may be a rare, low-grade malignant neoplasm, such as an adenosarcoma.

## Introduction

Uterine adenosarcoma is a mixed epithelial and mesenchymal tumor characterized by the proliferation of benign epithelial cells and malignant stromal cells. It often presents as a polypoid or exophytic lesion within the uterine cavity. Both obesity and diabetes mellitus have been identified as potential risk factors for its development [1]. Uterine adenosarcoma is a rare malignancy, accounting for approximately 5% of all uterine sarcomas [2]. The sarcomatous component is typically a low-grade endometrial stromal sarcoma, and the prognosis is relatively favorable when diagnosed at an early stage. However, accurate diagnosis is often challenging due to the limitations of pathological and imaging studies [3].

We report a case in which a lesion initially diagnosed as a submucosal uterine leiomyoma was treated by hysteroscopic tumor excision, leading to a final diagnosis of uterine adenosarcoma based on the histopathological evaluation.

## Case presentation

The patient was a 60-year-old nulliparous woman with a height of 157.5 cm, a weight of 72.6 kg, and a BMI of 29.3 kg/m². She experienced menarche at the age of 10 and menopause at the age of 50. Her medical history included hypertension and type 2 diabetes mellitus, which was well-controlled with oral DPP-4 inhibitors, maintaining an HbA1c level of 5.8%. She had no notable past medical history, and her family history was significant only for paternal lung cancer. The patient presented with irregular genital bleeding, which persisted for approximately one month, accompanied by lower abdominal pain. At her initial visit to our hospital, she underwent a transvaginal ultrasound, which also revealed a mass filling the uterine cavity. Magnetic resonance imaging (MRI) could not rule out the possibility of uterine malignancy, and the patient was referred to our department for further evaluation and treatment.

**Figure 1:**
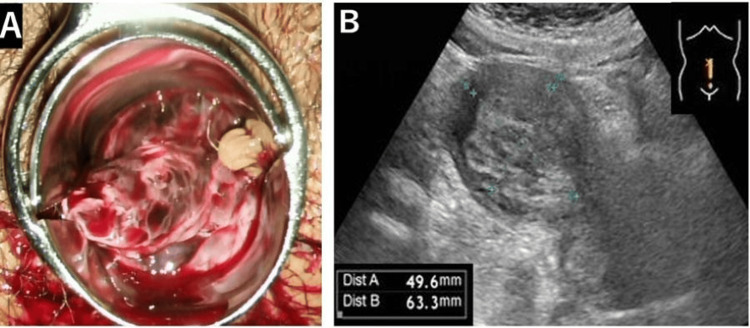
Findings on clinical examination. A) Speculum examination: a fragile, hemorrhagic mass containing small cystic components was observed prolapsing beyond the external cervical os into the vagina. B) Transabdominal ultrasound findings: a 50×63 mm mass containing small cystic components was observed within the uterine cavity.

At the initial examination, the uterus was the size of a clenched fist and exhibited good mobility. A fragile, hemorrhagic mass was observed prolapsing from the uterine cavity through the cervical canal, with part of the mass extending outside the vagina (Figure 1A). A transabdominal ultrasound revealed a honeycomb-like tumor measuring 50×63 mm, consisting of mixed small cystic and solid components within the uterine cavity (Figure 1B). No abnormalities were observed in the bilateral adnexa, and there was no evidence of ascites.

**Figure 2:**
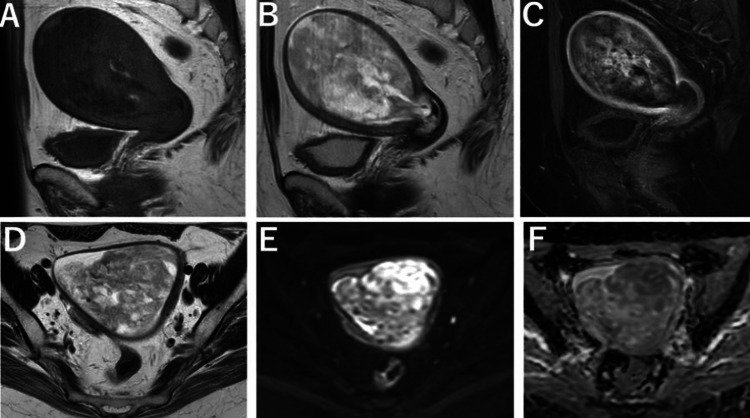
Findings on MRI. A) T1-weighted image (T1WI). B)T2-weighted image (T2WI): An 11×6 cm mass displaying mixed high signal intensity on T2-weighted images and isointense signal relative to the myometrium on T1-weighted images was observed, causing dilation of the internal cervical os.The mass contained cystic components. C) Fat-suppressed T1WI (40 seconds post-contrast) enhancement was observed in part of the tumor. D) T2WI. E) Diffusion-weighted image (DWI). F) Apparent diffusion coefficient (ADC) mild diffusion restriction was observed in part of the tumor.

The patient was afebrile; however, blood biochemical analysis revealed an elevated white blood cell (WBC) count of 17,000/µL (reference range: 4,000-11,000/µL) and a C-reactive protein (CRP) level of 7.53 mg/dL (reference range: <0.5 mg/dL), thus indicating an inflammatory response likely caused by infection at the extruded portion of the tumor. Renal function tests showed elevated blood urea nitrogen (BUN) at 27 mg/dL (reference range: 7-20 mg/dL) and creatinine (Cre) at 1.29 mg/dL (reference range: 0.5-1.2 mg/dL), also suggesting impaired renal function due to type 2 diabetes mellitus. Tumor marker levels included CA125 at 56 U/mL (reference range: <35 U/mL) and lactate dehydrogenase (LDH) at 184 U/L (reference range: 140-280 U/L). MRI revealed a tumor measuring 11×6 cm within the uterine cavity, characterized by mixed low and high signal intensities on T2-weighted images and low signal intensity on T1-weighted images. The tumor contained cystic components and demonstrated enhancement after contrast administration (Figures 2A, 2B, 2C). Diffusion-weighted imaging (DWI) showed mild diffusion restriction in certain parts of the tumor (Figures 2C, 2D, 2E).

No obvious pelvic lymph node enlargement was observed. Chest and abdominal CT scans also revealed no abnormalities. Cytological examinations, including cervical cytology, endometrial cytology, tumor biopsy, and endometrial biopsy, showed no evidence of malignancy.

**Figure 3:**
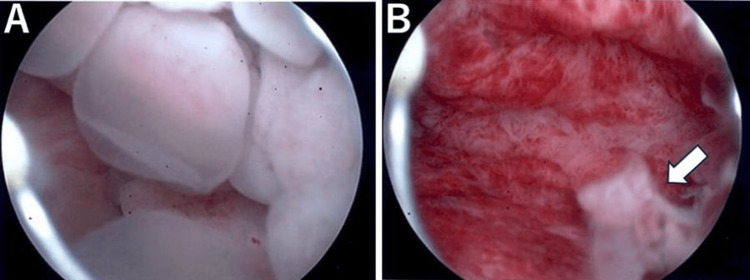
Findings on hysteroscopy. A) Before tumor resection: a soft, polypoid tumor with a smooth surface and multiple cysts was observed. B) After tumor resection: a stalk (arrow) was identified on the slightly left wall of the uterine body.

Given the patient’s strong desire to preserve her uterus and the preoperative diagnosis of a prolapsed leiomyoma, we opted to perform hysteroscopic tumor resection. Hysteroscopic findings revealed a normal appearance of the endocervical and endometrial surfaces, except for the tumor, which was pedunculated and attached to the left wall of the uterine body. The tumor was soft, pale yellow, and composed of numerous small cysts with a smooth surface occupying the uterine cavity (Figures 3A, 3B). Macroscopically, the lesion appeared distinct from typical uterine fibroids or polyps. The intrauterine tumor was removed as completely as possible using a sharp curette and placental forceps, and any remaining stalk was further resected under hysteroscopic guidance. The operation lasted 49 minutes, and blood loss was minimal.

**Figure 4:**
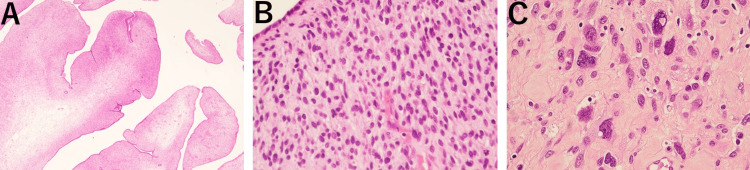
Histopathological findings. A) Hematoxylin and eosin stain (×40), periglandular cuffing, characterized by densely proliferated stromal components encasing the glandular structures, was observed. B) Hematoxylin and eosin stain (×100), the epithelial component displayed normal cells, while the stromal component showed mitotic figures. C) Hematoxylin and eosin stain (×400), the stromal component exhibited cells with nuclear enlargement and atypia.

Histopathological examination of the resected specimen revealed epithelial components with minimally atypical cells, while the stromal component exhibited nuclear enlargement and pleomorphism. Periglandular cuffing was also observed (Figures 4A, 4B, 4C).

**Figure 5:**
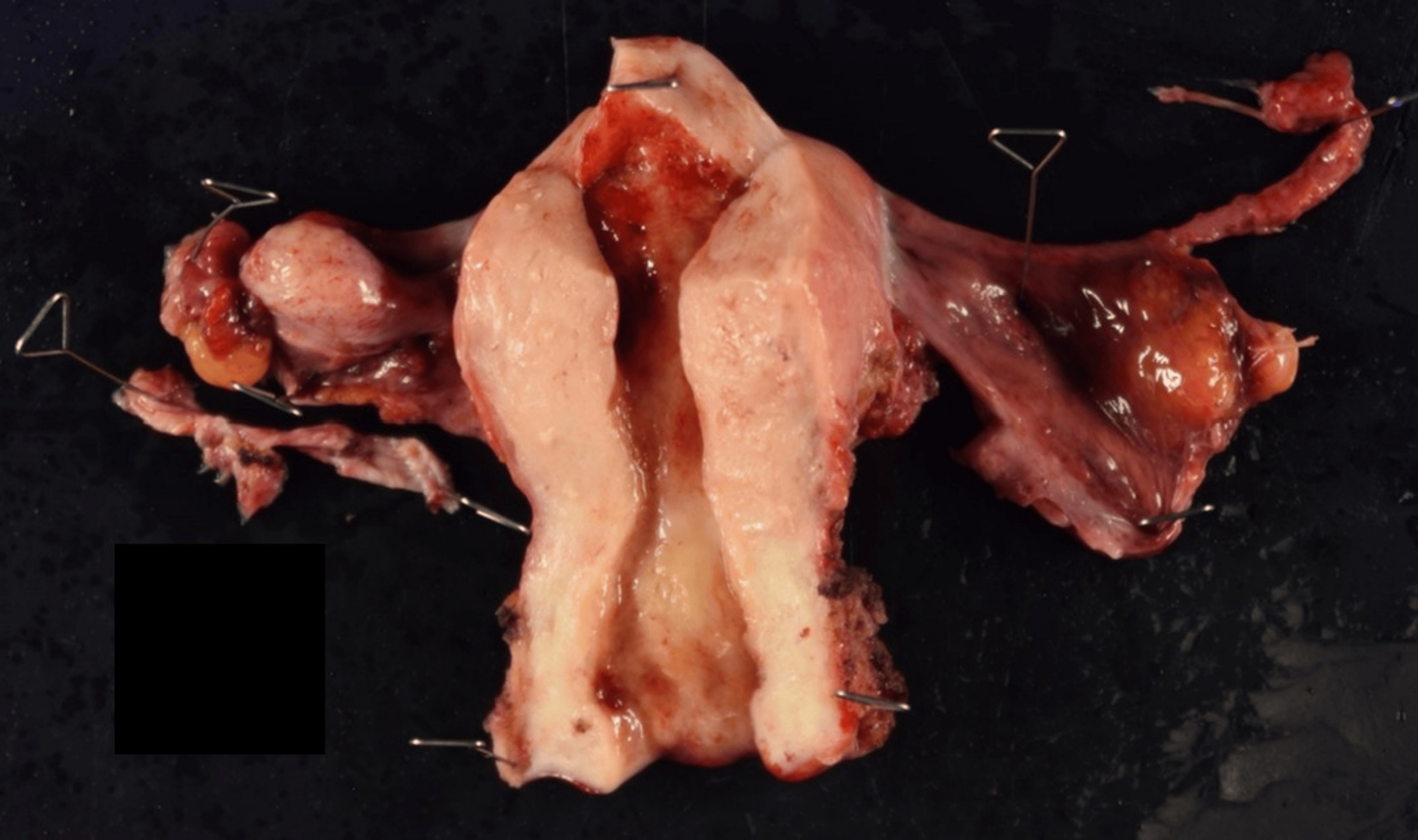
Surgical specimen. No macroscopic lesions were observed within the uterine cavity, and no malignant findings were identified histopathologically.

Immunohistochemical staining revealed partial positivity for CD10, while aSMA and h-caldesmon were negative. Sarcomatous overgrowth (SO), defined as the presence of high-grade sarcomatous components occupying more than 25% of the tumor, was not observed. Based on these findings, a diagnosis of uterine adenosarcoma was made. As an additional treatment, an abdominal simple total hysterectomy with bilateral salpingo-oophorectomy was performed, requiring two hours and 18 minutes with an estimated blood loss of 180 mL. Intraoperative findings revealed no ascites and no abnormalities in the uterus or bilateral adnexa. Visual and tactile examinations did not detect any obvious pelvic lymph node enlargement. The resected uterus was the size of a hen’s egg, with no apparent tumorous lesions in the uterine cavity, and no abnormalities were observed in the bilateral adnexa (Figure 5).

The peritoneal washing cytology was negative. Histopathological examination of the resected specimen showed no residual uterine adenosarcoma lesions on the endometrial surface and no evidence of myometrial invasion. Based on these findings, the diagnosis was TNM stage T1aN0M0 and clinical stage IA (FIGO 2008).

The postoperative course was uneventful, and the patient was discharged on the seventh postoperative day. Following standard protocols for malignancy follow-up, ultrasound examinations, and vaginal stump, cytology is performed every three months for the first two years after diagnosis. If any abnormalities are detected, additional imaging studies are conducted accordingly. At 18 months post-surgery, the patient remains under outpatient follow-up with no evidence of recurrence or metastasis.

## Discussion

Uterine adenosarcoma is a rare tumor, accounting for less than 0.2% of uterine malignancies and approximately 5% of uterine sarcomas [2]. It was first described by Clement et al. in 1974 [4]. The tumor commonly occurs in postmenopausal women, with reported median ages of 54 years (range: 15-84 years) [5] and 58 years (range: 14-89 years) [6]. Tate et al. reported a median age of 59 years (range: 12-88 years) in a study of 110 Japanese cases [3]. Risk factors for uterine adenosarcoma include tamoxifen use, prolonged estrogen replacement therapy, and pelvic radiation exposure [2], although none of these apply to the present case. It has also been reported that endometriosis may be associated with extrauterine adenosarcomas [2].

Sarcoma cells exhibit enhanced glucose uptake and metabolism, thus suggesting that metabolic processes play a critical role in tumor progression. Gluconeogenesis serves as a counter-regulatory pathway to glycolysis, and gluconeogenic enzymes may have important functions in the regulation of tumor cell proliferation. Among these, fructose-1,6-bisphosphatase 2 (FBP2), a key gluconeogenic enzyme, has been shown to be silenced in many subtypes of soft tissue sarcomas (STS), according to a study by Huangyang et al. [7]. Additionally, metabolomic analysis of leiomyosarcoma, synovial sarcoma, and liposarcoma has identified a comprehensive profile of 119 metabolites. Pathway enrichment analysis of these metabolites revealed significant enrichment in multiple metabolic pathways, including glycolysis, glutamate metabolism, and the tricarboxylic acid (TCA) cycle [8]. These findings suggest that metabolic alterations in tumors may contribute to tumor development and progression.

Similarly, metabolic abnormalities have been implicated in the initiation and progression of uterine adenosarcoma [9,10]. Diabetes mellitus has been reported as one of the risk factors for uterine sarcomas [1]. Previous case reports have documented comorbid conditions such as diabetes, hypertension, and obesity in patients with uterine adenosarcoma. It has been suggested that these metabolic disorders may contribute to tumor development through mechanisms such as abnormal estrogen metabolism and chronic inflammation [11]. However, there is currently no definitive evidence to establish a direct causal relationship between diabetes and uterine adenosarcoma. A search was conducted for case reports and review articles on uterine adenosarcoma published between 2014 and 2024, focusing on those documenting comorbidities, including diabetes mellitus. The findings are summarized in Table 1 [12,13].

**Table 1:** Clinical characteristics and treatment outcomes of uterine adenosarcoma cases.

Case	Age	Stage (FIGO)	Menopausal status	Comorbidities/past history	Symptoms	Treatment	References
Case1	62	Stage IB	Postmenopausal	Diabetes, hypertension/breast cancer	Pelvic mass	Surgery and radiotherapy	[12]
Case2	64	Stage IV (liver metastasis)	Postmenopausal	Diabetes, hypertensive cardiovascular disease	General malaise, abdominal pain, vaginal bleeding	Surgery only	[13]

These reports suggest that metabolic disorders, such as diabetes, hypertension, and obesity, may be associated with the development and progression of uterine adenosarcoma. In the present case, the patient had comorbid obesity and type 2 diabetes mellitus, which may have been associated with the formation and progression of the tumor. These findings suggest that managing metabolic disorders, such as diabetes and obesity, may play an important role in risk management and preventive strategies for uterine adenosarcoma.

The most common site of tumor origin is the endometrium, accounting for 87% of cases, followed by the endocervical lining (9%) and the myometrium (4%) [6]. Consequently, uterine adenosarcoma often begins as a surface polyp on the endometrium and later develops into an exophytic lesion infiltrating the endometrium. Macroscopically, it frequently resembles a large endometrial polyp or a submucosal leiomyoma [14]. The cut surface of uterine adenosarcoma typically appears yellowish brown, with areas of hemorrhage and necrosis, and is considered a low-grade malignancy. The median tumor size has been reported to be 5 cm (range: 1-17 cm) [2]. The tumor often originates in the uterine fundus, occupying the uterine cavity and sometimes extending to the external cervical os. Caroll et al. reported that in 27 out of 74 cases (36%), the tumor prolapsed through the external cervical os [5]. Uterine adenosarcoma is more commonly observed in Caucasian women (69%), with the most frequent symptom being abnormal vaginal bleeding, occurring in 65-75% of cases. Other symptoms include pelvic pain and a palpable mass [2]. In this case, the patient presented with postmenopausal vaginal bleeding and lower abdominal pain. At the initial visit following referral to our hospital, a polypoid tumor measuring 11 cm in diameter was observed originating from the endometrium of the left uterine wall, extended through the cervical canal, and partially prolapsed outside the vagina.

Pathological examination revealed characteristic periglandular cuffing, thus indicative of a phyllodes tumor-like structure. The stromal component consisted of proliferating endometrial stromal cells, with findings of increased cellular atypia and mitotic activity. In such cases, the degree of atypia varies, with mild atypia observed in 61% of cases, moderate atypia in 26%, and severe atypia in 9% [2]. Immunohistochemical staining typically shows positivity for various cytokeratins in the epithelial component, while the stromal component frequently exhibits positivity for CD10, estrogen receptor (ER), and progesterone receptor (PR). Smooth muscle markers such as desmin and h-caldesmon are also often positive [15]. According to Tate et al., 76% of cases are diagnosed as malignant based on biopsy pathology, and 33% are specifically diagnosed as adenosarcoma. In 14% of cases, repeated biopsies yield a diagnosis of benign tumor [3]. The difficulty in achieving a diagnosis through biopsy pathology is attributed to the nature of adenosarcoma, which consists of benign epithelial components and low-grade sarcomatous components. When biopsy samples are taken from areas with minimal atypia, the tumor may be underestimated and misdiagnosed as a benign lesion. In such cases, the tumor is often diagnosed as an adenofibroma or polyp, with some cases being misdiagnosed as recurrent polyps. In the present case, the biopsy specimen obtained during the outpatient examination showed no evidence of malignancy, and a diagnosis of adenosarcoma was not established then.

The ultrasonographic appearance of uterine adenosarcoma is characterized by a heterogeneous thickened endometrium with small cystic components within the uterine cavity [16].

MRI features of uterine adenosarcoma include an intracavitary tumor that expands the uterine cavity, thus causing thinning and stretching of the myometrium. The tumor often appears heterogeneous and lobulated, sometimes extending from the external cervical os into the vagina. Due to its polypoid nature, it typically has well-defined margins and is often pedunculated at its base. On T1 and T2-weighted images, the tumor may exhibit a higher signal intensity compared to the myometrium. The solid components within the tumor exhibit an intermediate signal intensity on both T1 and T2-weighted images, while multiple small cystic areas display a low signal intensity on T1-weighted images and a high signal intensity on T2-weighted images. Necrosis and hemorrhage are commonly observed within the tumor [17].

Regarding diffusion-weighted imaging (DWI), restricted diffusion is not always present [18]. Based on imaging findings alone, a differential diagnosis may include degenerative uterine leiomyoma, endometrial polyp, carcinosarcoma, or gestational trophoblastic disease [19]. According to Tate et al., only 1 out of 110 cases is diagnosed as adenosarcoma based on imaging studies [3]. Takeuchi et al. concluded that while adenosarcomas often resemble benign tumors on imaging, the presence of scattered small cysts within the tumor on MRI may be a key finding suggestive of this rare tumor [18]. In the present case, MRI revealed an intracavitary tumor with a low signal intensity on T1-weighted images and mixed low and high signal intensities on T2-weighted images, accompanied by small cystic lesions. Mild diffusion restrictions were also observed.

There have been several reported cases of uterine adenosarcoma diagnosed following hysteroscopic tumor excision [14]. In the present case, although malignancy could not be ruled out based on MRI findings, no definitive malignant features were observed. Preoperative evaluations, including cervical cytology, endometrial biopsy, and tumor biopsy, also revealed no evidence of malignancy. Consequently, the tumor was preoperatively diagnosed as a submucosal leiomyoma. However, a definitive diagnosis of uterine adenosarcoma was established through hysteroscopic tumor excision.

Uterine adenosarcoma is often detected at an early stage, primarily because abnormal genital bleeding is a common presenting symptom, as previously mentioned. According to Caroll et al., a study of 74 cases reported that 80% were stage I, 14% were stage II, 4% were stage III, and 2% were stage IV [5].

Prognostic factors for uterine adenosarcoma include myometrial invasion, extrauterine spread, the presence of sarcomatous overgrowth (defined as sarcomatous components occupying more than 25% of the tumor), advanced age, and lymphovascular invasion [2,20]. Lymph node metastasis and distant metastasis are rare, occurring in 3.1% and 2.7% of cases, respectively [21]. Risk factors for lymph node metastasis include myometrial invasion, sarcomatous overgrowth, and tumor size [22]. Recurrence rates are 36% in cases with myometrial invasion, compared to 7% in cases without myometrial invasion [5].

The 5-year survival rates for uterine adenosarcoma are 79% for stage I, 69% for stage II, 48% for stage III, and 15% for stage IV, which are relatively favorable compared to other sarcomas. For example, the corresponding survival rates for stage I, II, Ⅲand Ⅳ carcinosarcomas are 51%, 33%, 24%, and 9%, respectively [20].

There is no established standard treatment for uterine adenosarcoma; however, a hysterectomy with a bilateral salpingo-oophorectomy is recommended. In 81% of cases, a bilateral adnexectomy was performed alongside a hysterectomy, with adnexal metastases reported in 17% of cases and ovarian metastases in 85% of those cases. Lymphadenectomy is generally not recommended due to the low rate of lymph node metastasis [2,14]. Although limited to case reports, in young women desiring fertility preservation, tumor resection alone or in combination with chemotherapy has been reported as an option when the tumor is small and when there is no sarcomatous overgrowth or myometrial invasion [6]. Successful childbirth has been achieved in some cases; however, standard surgical treatment is recommended after childbirth. Postoperative radiotherapy has not been shown to be effective, and no established chemotherapy regimen has proven to be consistently effective. The efficacy of postoperative chemotherapy for uterine adenosarcoma remains unclear. However, some reports have suggested the effectiveness of combination regimens, such as doxorubicin with ifosfamide and gemcitabine with docetaxel. Additionally, cases with estrogen receptor (ER) or progesterone receptor (PgR) positivity have been treated with hormonal therapies, such as medroxyprogesterone acetate (MPA), with reported success [2].

Recurrent uterine adenosarcoma is most often localized to the vagina, pelvis, or abdomen [6,23]. Even at recurrence, lymph node metastases and distant metastases are rare. When distant metastases occur, the most common sites are the lungs and liver. Late recurrences are also frequent, with approximately one-third of recurrences reported to occur five years or more after surgery [24]. For local recurrences, surgical resection is recommended, while for distant metastases, surgical resection followed by the aforementioned chemotherapy regimens should be considered [2].

Adenosarcoma is an extremely rare tumor, thus making it impractical to conduct clinical trials for treatments other than surgery. Consequently, it is challenging to establish treatment strategies based on robust evidence.

## Conclusions

Uterine adenosarcoma typically presents as a polypoid exophytic lesion and is often misdiagnosed as an endometrial polyp or submucosal leiomyoma on MRI. It is also challenging to diagnose through biopsy-based pathological examination. In postmenopausal women with diabetes mellitus who present with persistent abnormal genital bleeding and a polypoid lesion occupying the uterine cavity, the possibility of low-grade malignancies, such as uterine adenosarcoma, should be considered, even in the absence of overt malignant findings. In such cases, it is essential to perform a pathological diagnosis through hysteroscopic tumor excision rather than relying solely on biopsy.
